# Dynamic PET/CT Imaging of ^68^Ga-FAPI-04 in Chinese Subjects

**DOI:** 10.3389/fonc.2021.651005

**Published:** 2021-03-11

**Authors:** Shuailiang Wang, Xin Zhou, Xiaoxia Xu, Jin Ding, Teli Liu, Jinquan Jiang, Nan Li, Hua Zhu, Zhi Yang

**Affiliations:** ^1^Institute of Medical Technology, Peking University Health Science Center, Beijing, China; ^2^Key Laboratory of Carcinogenesis and Translational Research (Ministry of Education/Beijing), Key Laboratory for Research and Evaluation of Radiopharmaceuticals (National Medical Products Administration), Department of Nuclear Medicine, Peking University Cancer Hospital & Institute, Beijing, China

**Keywords:** dynamic PET/CT, fibroblast activation protein, ^68^Ga-FAPI-04, lung cancer, variability

## Abstract

This study aims to further explore dynamic ^68^Ga-FAPI-04 PET/CT imaging of healthy Chinese subjects and lung cancer patients. Moreover, the variability of ^68^Ga-FAPI-04 uptake in normal organs was measured to provide a basis for analyzing its biological distribution, interpreting auxiliary images, determining the reliability of image quantification, and monitoring treatment. Six patients (3 subjects without tumors and 3 lung cancer patients) separately underwent ^68^Ga-FAPI-04 and 2-[^18^F]FDG PET/CT imaging within 1 week. The biodistribution and internal radiation dosimetry were reported and compared with data previously obtained from Caucasian patients. Moreover, the mean SUV (standardized uptake value) was normalized to body mass or to lean body mass (SUL), and the coefficients of variation (CVs) were calculated and compared for each volume of interest. The average whole-body effective dose was calculated to be 1.27E-02 mSv/MBq, which was comparable with previously reported results of ^68^Ga-FAPI-04 probes. Furthermore, the SUVmean was slightly higher than the SULmean in most organs; however, the CV of the SULmean for most organs was higher than that of the SUVmean at later time points. In the liver, the CV of the SUVmean was lower (12.7%) than that of the SULmean and was similar to the CV for corresponding 2-[^18^F]FDG PET/CT value (11.8%). In addition, ^68^Ga-FAPI-04 PET/CT showed good efficacy for diagnosing lung cancer patients in this study. A comparison of the radiation dosimetry obtained before from a Caucasian population demonstrated no clinically significant differences between these two populations after ^68^Ga-FAPI-04 injection. The variability in most organs was slightly lower for SUVmean than for SULmean, suggesting that SUVmean may be the preferable parameter for quantifying images obtained with ^68^Ga-FAPI-04. In addition, ^68^Ga-FAPI-04 PET/CT imaging is expected to be a promising tool for diagnosing lung cancer.

## Introduction

Fibroblast activation protein (FAP), or seprase, is a type II transmembrane glycoprotein that possesses serine protease activity and is closely associated with the invasion and metastasis of human cancers (1). The expression of FAP in normal tissue is highly restricted, but this protein becomes overactive in the tumor stroma, which comprises many cancer-associated fibroblasts (2). Furthermore, the expression of FAP is associated with poor prognosis in several kinds of cancer (3–5). Hence, FAP is a promising specific theranostic target for cancer.

In recent years, a variety of small-molecule inhibitors of FAP, or FAPIs, have been synthesized, and their antitumor activity has been studied in detail (6–8). Among these inhibitors, (4-quinolinoyl)glycyl-2-cyanopyrrolidine-based FAPIs fabricated by Koen et al. showed the highest affinity for FAP and were further modified and developed by scientists into cancer theranostic probes (9). The application of radiolabeled ^68^Ga-FAPI-04 for PET/CT imaging in patients with breast cancer was first demonstrated by Lindner et al. in 2018 (10). More recently, the application of ^68^Ga-FAPI-04 PET/CT was further extended to the clinical detection of 28 different kinds of cancer (11, 12). Moreover, the potential of FAPIs for cancer detection and radionuclide therapy has been fully revealed in several recent studies (13, 14).

To the best of our knowledge, no previous study has focused on dynamic PET/CT imaging with ^68^Ga-FAPI-04 in Chinese patients. Furthermore, the intrinsic variability of ^68^Ga-FAPI-04 distribution in normal organs needs to be investigated before the ability of this probe to detect and monitor different types of cancer can be evaluated; head-to-head comparisons between ^68^Ga-FAPI-04 PET/CT and 2-[^18^F]FDG PET/CT in both healthy volunteers and cancer patients are lacking. Therefore, this study aims to further evaluate dynamic imaging with ^68^Ga-FAPI-04 in healthy patients and cancer patients. The clinical safety, biodistribution and internal radiation dosimetry assessments of ^68^Ga-FAPI-04 have been performed previously with a Caucasian cohort of patients (11), and these data were compared with the results obtained from this work. Because ethnicity may affect the expression of transporters and metabolizing enzymes that determine pharmacokinetics and biodistribution, it is essential to confirm interethnic comparability for global use of this PET drug.

## Materials and Methods

### Subjects

This study was approved by the Ethics Committee of Peking University Cancer Hospital (2019 KT95), and registered in Chinese Clinical Trial Registry (ChiCTR2000038080). Written informed consent was obtained from each participant prior to ^68^Ga-FAPI-04 PET/CT imaging. The inclusion criteria included the following: age older than 18 years, ability to provide informed written consent, and normal liver and kidney function. The exclusion criteria included the following: liver and renal dysfunction and pregnancy or current lactation, and patients diagnosed with wound healing, arthritis, inflammation or cirrhosis liver. Finally, two groups of subjects (three subjects without tumors and three lung cancer patients) were enrolled in this study. The demographic data of these subjects are shown in Table 1.

**Table 1:** The demographic data of all subjects.

**Patient No**.	**Sex**	**Age (y)**	**Weight (kg)**	**Injected activity (MBq)**	**Pathological type**
1	F	53	60	129.5	Lung Ca.
2	F	62	73	133.2	No tumor
3	F	33	47	118.4	No tumor
4	M	62	75	103.2	Lung Ca.
5	M	53	80	150.6	No tumor
6	M	63	80	257.5	Lung Ca.

### Radiopharmaceuticals

^68^Ga-FAPI-04 was synthesized through the modified method described in previously reported literature (10). Briefly, ^68^GaCl_3_ (3 mL, 0.05 M HCl) eluted from a ^68^Ge-^68^Ga generator (1.85 GBq, ITG Co., Ltd, Germany) and 20 μg of the DOTA-FAPI-04 (HUAYI TECHNOLOGY Co., Ltd, China) precursor were mixed. The reaction was carried out at 95°C for 10 min after the pH was adjusted to 4.0 by sodium acetate. The radiochemical purity of the final product was tested by radio-HPLC. Before intravenous administration to the patients, the ^68^Ga-labeled product was purified by solid-phase extraction with Sep-Pak Light C18 (Waters, USA), diluted with saline and further processed with a polytetrafluoroethylene filter (0.2 μm).

### Toxicity Monitoring

All subjects were monitored by a medical technician between tracer application and up to 30 min after the PET/CT examination was finished. During this period, all subjects were asked to report any abnormalities.

### Examination Procedures

No specific preparation was required for any of the subjects on the day of ^68^Ga-FAPI-04 PET/CT scanning. A low-dose CT scan (120 kV, 35 mA, slice: 0.6 mm, matrix: 512 × 512) was performed before the ^68^Ga-FAPI-04 injection; then, a whole-body dynamic PET scan was performed immediately after the intravenous injection of ^68^Ga-FAPI-04 for all subjects and continued for 6 frames (frame 1, 5 mm/s; frame 2, 2 mm/s; frame 3, 2 mm/s; frame 4, 1.5 mm/s; frame 5, 1.5 mm/s; and frame 6, 1 mm/s), covering a period up to 60 min after the injection. All subjects underwent a follow-up 2-[^18^F]FDG PET/CT static scan within a week after ^68^Ga-FAPI-04 imaging. The ^68^Ga-FAPI-04 and 2-[^18^F]FDG PET/CT scans were acquired using a Biograph mCT Flow 64 scanner (Siemens, Erlangen, Germany) [120 kV, 146 mAs, slice: 3 mm, matrix: 200 × 200, full width at half maximum (FWHM): 5 mm, filter: Gaussian, field of view (FOV): 256 (head), 576 (body)], which continuously moved the patient bed to cover the entire body of each subject (from the top of the skull to the middle of the femur).

### Data Analysis

A Siemens workstation (syngo.via Client 4.1) was used for postprocessing. Two experienced nuclear medicine physicians independently reviewed all images, and any discordant results were resolved by consensus. To analyze the biodistribution and variability of ^68^Ga-FAPI-04, volumes of interest (VOIs) were manually drawn on a section of each major organ/tissue within the edges of each organ. Data, including SUVmax, SUVmean, SULmax, SULmean, and average activity concentration (Bq/mm^3^), were obtained to determine the organ biodistribution and to calculate the human organ dosimetry. The dynamic PET/CT images and biodistribution of ^68^Ga-FAPI-04 in KM mice were also analyzed as described in the Supplementary Information.

For dosimetry analysis, heart content, lung, liver, spleen, pancreas, kidneys, uterus, urinary bladder content and body remainder were selected as source organs. The volumes of the source organs on CT images were calculated, and their mean counts/mL (kBq/mL) were determined from PET images at different time points. Whole-organ activity (MBq) was calculated by multiplying organ volume by mean counts/mL and dividing by 1,000. The percentage of injected activity was calculated to generate the activity concentration-time curve of each source organ. Biexponential curve fitting was applied to activity concentration-time curves of the uterus and urinary bladder content, while monoexponential curve fitting was applied to other source organs, to calculate the areas under the curve (AUCs), and human organ dosimetry was further estimated using OLINDA/EXM software (version 2.0; Hermes Medical Solutions AB). Organ-absorbed doses and total effective doses, as well as residence time for each patient, were obtained using reference adult male and female models.

For the SUVs within the tumor lesions, an elliptical VOI was placed over each metabolically active lesion that was suspected to be malignant with a 50% threshold isocontour. Compared to the uptake in the adjacent normal tissue, focal uptake in the tumor lesion was considered positive. To compute the tumor-to-non-tumor SUV ratio (SUR), we obtained the non-tumor SUV from a disease-free area within the surrounding or contralateral normal tissue.

### Statistical Analysis

In our study, all statistical analyses were conducted using SPSS software (version 25.0; IBM Corp.). The data are presented as the mean ± SD for each organ. The paired samples *T*-test was used to compare differences between SUVmean and SULmean. *P* < 0.05 were considered statistically significant.

## Results

### Tracer Administration and PET/CT Imaging

**Figure 1:**
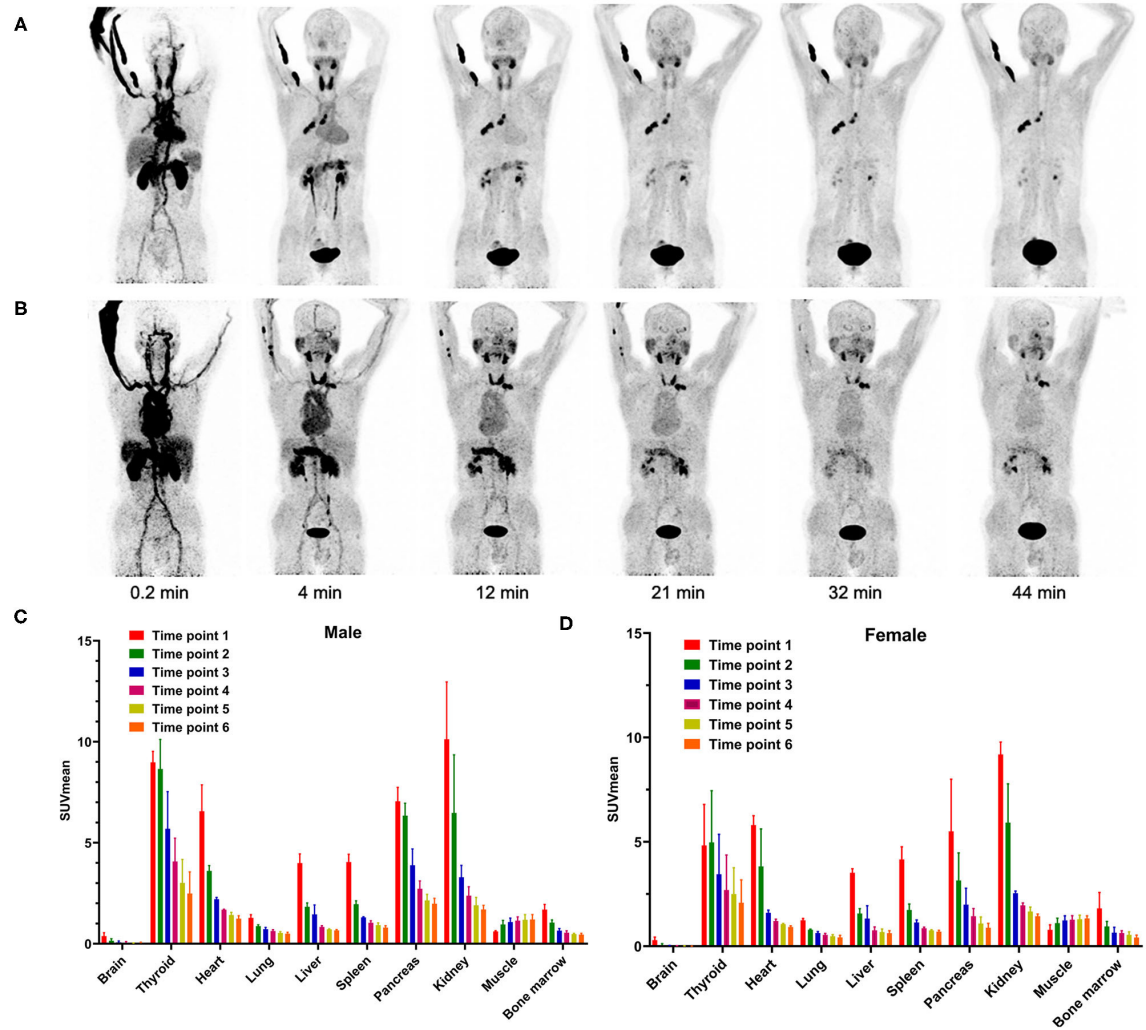
Representative PET MIP images of **(A)** female patient 1 and **(B)** male patient 4 at different time points. Dynamic ^68^Ga-FAPI-04 PET/CT-based biodistribution analysis of **(C)** male and **(D)** female Chinese subjects.

^68^Ga-FAPI-04 was achieved with high radiolabeling yield of over 90%, and the radiochemical purity was higher than 99%, as detected by radio-HPLC. Furthermore, the specific activity of radiopharmaceuticals applied in this study was calculated to be 32.2–48.3 MBq/nmol. All patients received a bedside administration of ^68^Ga-FAPI-04 with an injected activity ranging from 103.2 to 257.5 MBq. No adverse effects were reported by any subject during the whole imaging period. The representative dynamic PET maximum intensity projections (MIPs) of subject 1 (female) and subject 4 (male) are shown in Figures 1A,B. The PET image of a representative healthy volunteer is presented in Supplementary Figure 1.

### Biodistribution

**Figure 2:**
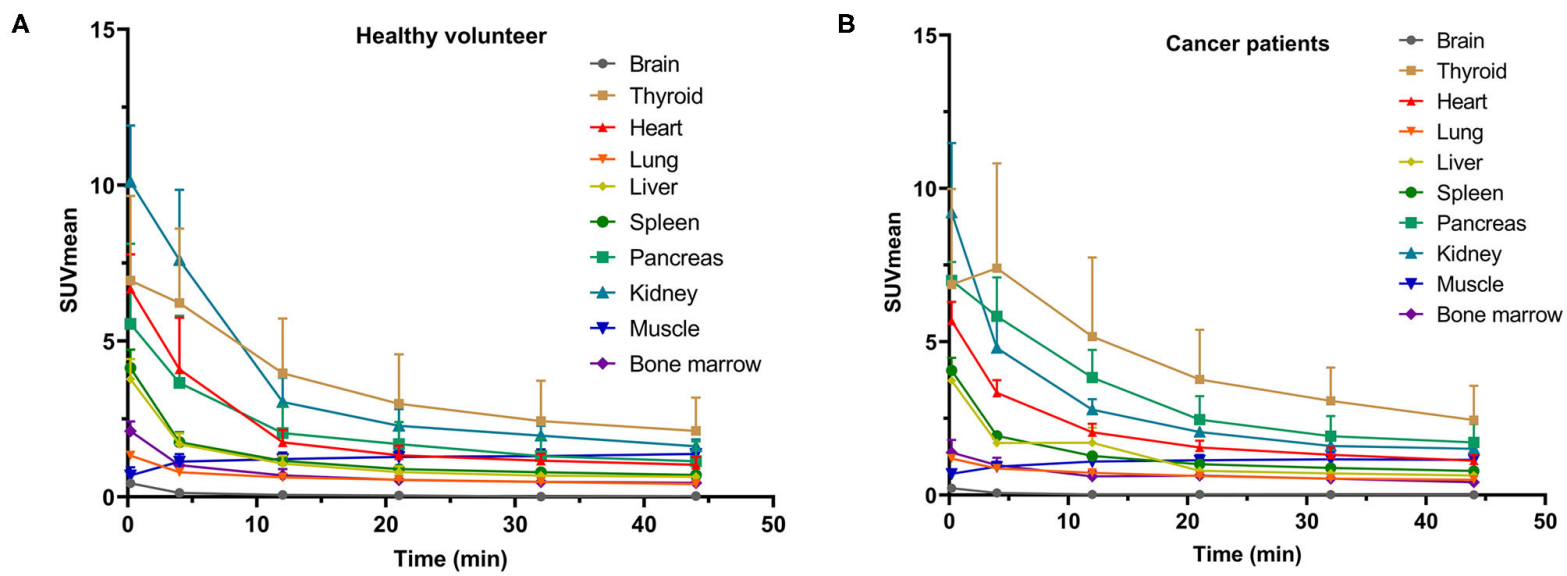
SUVmean of various organs on dynamic ^68^Ga-FAPI-04 PET/CT for **(A)** subjects without tumors and **(B)** cancer patients at different time points.

**Figure 3:**
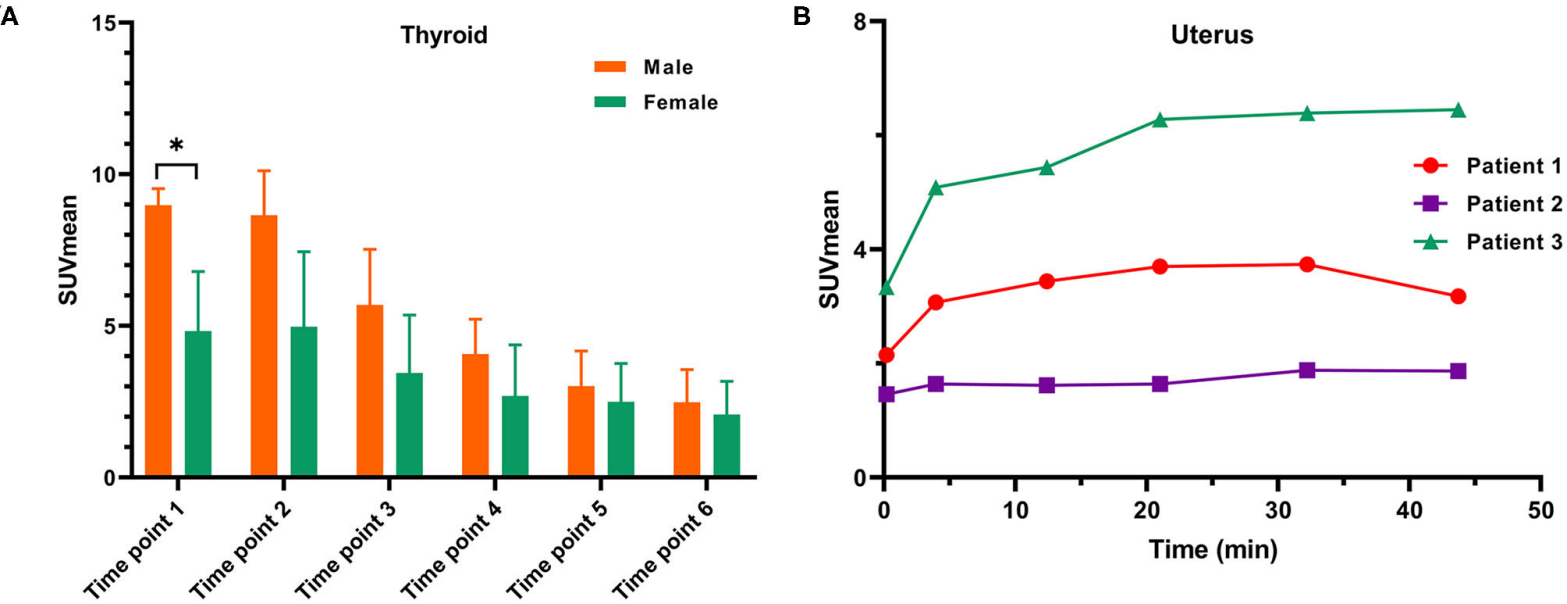
**(A)** A comparison of SUVmean variation on dynamic ^68^Ga-FAPI-04 PET/CT in the thyroids of male and female subjects and **(B)** SUVmean measurements of the uterus in three female subjects on dynamic ^68^Ga-FAPI-04 PET/CT. *p < 0.05.

**Figure 4:**
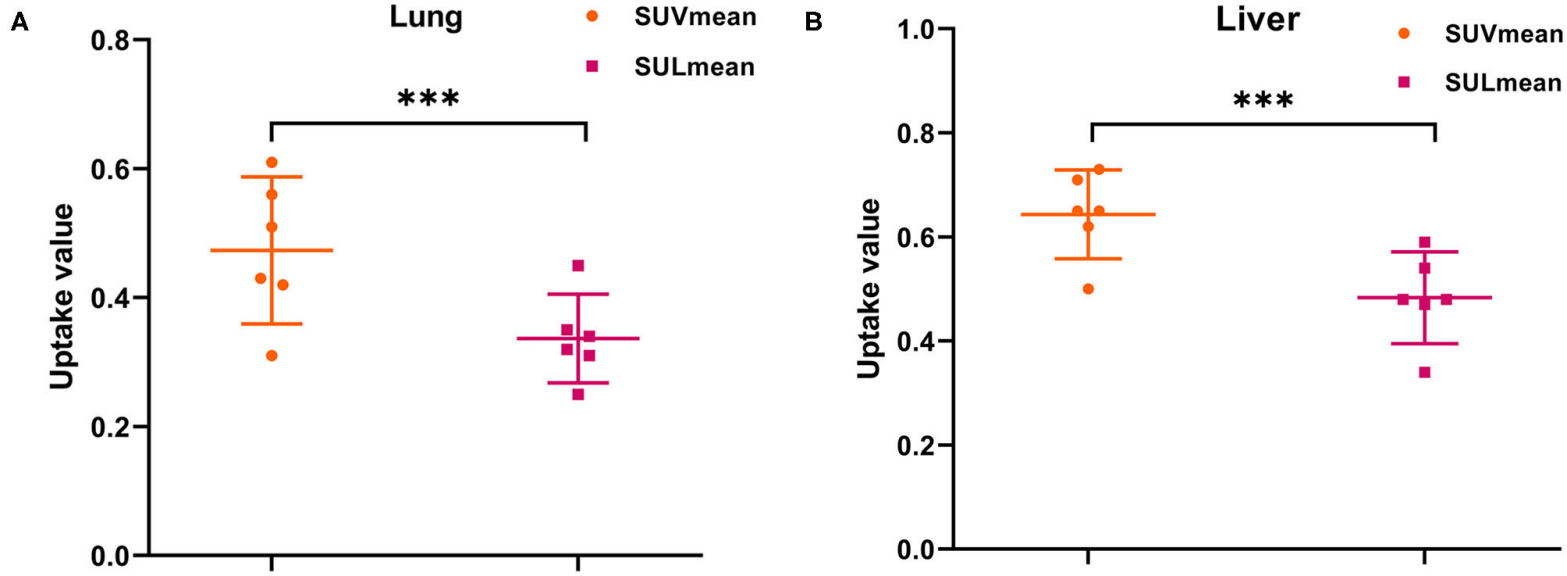
Comparison of SUVmean with SULmean for the **(A)** lung and **(B)** liver across all subjects at time point 6 (mean with 95% CI). ***p < 0.001.

In our study, the SUVmean and SUVmax measurements of the organs of interest at six different time points (0.2 min, 4 min, 12 min, 21 min, 32 min, 44 min) were measured and summarized. The *in vivo* biodistribution of ^68^Ga-FAPI-04 in male and female subjects is presented in Figures 1C,D. According to the PET maximum intensity projections (MIPs), the blood pool in these patients was clearly visualized at early time points but quickly vanished over time. The submaxillary glands and parotid glands showed physiological uptake, while the uteruses of female patients remained apparent during the whole imaging period. In most organs, except for muscle and uterus, the SUVmean decreased over time. Radio signals in the bladders of patients increased as early as 4 min postinjection, and the highest SUVmean measurements were observed in the bladder at later time points because of the rapid renal excretion of the tracer. The average organ SUVmean variation over time in healthy volunteers and cancer patients is presented in Figure 2. The SUVmean in most organs decreased with time, and there were no differences between healthy volunteers and cancer patients. Interestingly, we found that the SUVmean of the thyroid in male patients was higher than that in female patients at earlier time points (as shown in Figure 3A), while the SUVmean of the uterus in female patients increased slowly within 1 h postinjection (Figure 3B). Furthermore, the SUVmean and SULmean of ^68^Ga-FAPI-04 in each organ were compared to investigate the variability in normal organ uptake. As shown in Tables 2, 3, the SULmean was slightly lower than the SUVmean in most organs across all patients at time point six. However, the CV of the SULmean for most organs was higher than that of the SUVmean. The comparison of SUVmean and SULmean in the lung and liver across all patients at time point 6 is presented in Figure 4. Similar to the dynamic images of humans, the dynamic ^68^Ga-FAPI-04 PET/CT images of KM mice indicated that ^68^Ga-FAPI-04 was cleared rapidly from the body (Supplementary Figure 2). In the biodistribution analysis, almost all normal organs of KM mice showed low uptake of ^68^Ga-FAPI-04, and the uptake reached a plateau within 30 min. However, the organs that showed the highest uptake of ^68^Ga-FAPI-04 were bone and muscle. The uptake value of ^68^Ga-FAPI-04 in the muscle of mice remained stable within the detection period. In contrast to the results from the human study, ^68^Ga-FAPI-04 uptake in the bone of mice increased over time (Supplementary Figure 3), which has not been revealed in any previous study.

**Table 2:** SUVmean and SULmean of each organ across all subjects at time point 6.

**Organ**	**SUVmean**	**SULmean**	***p*-value**
Thyroid	2.3 ± 1.0	1.7 ± 0.8	0.005
Heart	1.1 ± 0.2	0.8 ± 0.2	0.000
Lung	0.5 ± 0.1	0.3 ± 0.1	0.001
Liver	0.6 ± 0.1	0.5 ± 0.1	0.000
Spleen	0.7 ± 0.1	0.6 ± 0.1	0.000
Pancreas	1.4 ± 0.6	1.1 ± 0.5	0.001
Kidney	1.6 ± 0.2	1.2 ± 0.2	0.000
Parotid	1.9 ± 0.5	1.5 ± 0.4	0.000
Muscle	1.3 ± 0.2	1.0 ± 0.1	0.001
Bone marrow	0.4 ± 0.1	0.3 ± 0.1	0.000

**Table 3:** Coefficient of variation of the SUVmean and SULmean for each organ across all subjects at time point 6.

**Organ**	**CV of SUVmean (%)**	**CV of SULmean (%)**
Thyroid	43.6	43.8
Heart	18.9	21.9
Lung	19.6	19.5
Liver	12.7	17.4
Spleen	12.3	16.9
Pancreas	44.6	47.3
Kidney	13.1	16.7
Parotid	28.0	30.1
Muscle	14.9	12.1
Bone marrow	20.1	24.9

### The Differences in CV Between ^68^Ga-FAPI-04 and 2-[^18^F]FDG in Normal Organs

The calculated CVs of the SUVmean measurements of 17 organs/tissues on ^68^Ga-FAPI-04 and 2-[^18^F]FDG images are shown in Table 4. The lungs had the lowest variability in ^68^Ga-FAPI-04 uptake of the studied organs with a CV of 12.2%, whereas the bone cortex had the highest variability (96.3%). The CV of liver uptake on ^68^Ga-FAPI-04 images was 12.4%; for reference, the CV of the liver with 2-[^18^F]FDG was 11.8%. The CV of ^68^Ga-FAPI-04 uptake in the pancreas was 42.2%, which was higher than that of 2-[^18^F]FDG (12.4%).

**Table 4:** Comparison of the CVs of SUVmean measurements between ^68^Ga-FAPI-04 and 2-[^18^F]FDG PET/CT.

Organ^*^	**FAPI-CV(%)**	**FDG-CV(%)**
Parotid	31.4	31.6
Thyroid	40.1	33.2
Heart	17.3	11.7
Lungs	12.2	21.8
Liver	12.4	11.8
Gallbladder	23.9	53.2
Pancreas	42.2	12.4
Spleen	13.5	14.4
Kidney	14.4	9.6
Bone marrow	19.1	16.8
Bone cortex	96.3	52.7
Stomach	18.6	37.1
Small intestine	22.0	25.4
Colon	56.8	36.7
Muscle	16.3	12.0

* *n = 5 (^18^F-FDG imaging of the third subject was not obtained)*.

### Comparison of Internal Radiation Dosimetry With a Caucasian Population

The pooled subject dosimetry results from OLINDA/EXM 2.0 are shown in Table 5, and the results for each subject are shown in Supplementary Table 1. In this study, the urinary bladder wall showed the highest average absorbed dose, followed by the uterus, kidneys, lungs, spleen, heart wall, pancreas, rectum and ovaries. The absorbed doses of other organs were below 8.12E-03 mGy/MBq. The organ with the highest average effective dose was also the urinary bladder wall, followed by the lungs, stomach wall and red marrow, as shown in Supplementary Table 2. The average whole-body effective dose was calculated to be 1.27E-02 mSv/MBq, which was comparable with previously reported results of ^68^Ga-FAPI-04 probes (11) (Table 5). Thus, the administration of 100 MBq ^68^Ga-FAPI-04 would result in a 1.27 mSv whole-body effective dose. Together with an approximately 3.7 mSv from one low-dose CT attenuation scan (15), a single ^68^Ga-FAPI-04 PET/CT imaging procedure would result in an estimated total effective dose of 4.97 mSv in adult Chinese subjects. Furthermore, the residence times of the source organs in each subject are summarized in Supplementary Table 3.

**Table 5:** Mean organ-absorbed doses (mGy/MBq) and whole-body effective doses (mSv/MBq) were compared between Chinese and Caucasian subjects.

**Target organ**	**Chinese (this work)**	Caucasian^*^
Adrenals	8.12E-03	1.12E-02
Brain	5.34E-03	9.11E-03
Breasts	5.73E-03	8.88E-03
Esophagus	6.27E-03	/
Eyes	5.35E-03	/
Gallbladder wall	6.72E-03	1.13E-02
Left colon	6.83E-03	1.17E-02
Small intestine	6.99E-03	1.13E-02
Stomach wall	6.72E-03	1.06E-02
Right colon	6.60E-03	1.11E-02
Rectum	1.07E-02	/
Heart wall	1.15E-02	2.02E-02
Kidneys	2.16E-02	4.43E-02
Liver	7.57E-03	1.46E-02
Lungs	1.60E-02	9.89E-03
Ovaries	1.02E-02	1.19E-02
Pancreas	1.14E-02	1.13E-02
Prostate	7.58E-03	/
Salivary glands	5.66E-03	/
Red marrow	6.28E-03	2.08E-02
Osteogenic cells	4.99E-03	2.16E-02
Spleen	1.18E-02	1.05E-02
Testes	5.60E-03	1.01E-02
Thymus	6.67E-03	1.01E-02
Thyroid	5.94E-03	9.82E-03
Urinary bladder wall	1.45E-01	9.91E-02
Uterus	4.03E-02	1.30E-02
Total body	7.28E-03	1.90E-02
Effective dose	1.27E-02	1.64E-02

* *Calculated from the data of reference (11)*.

### Characteristics of Dynamic ^68^Ga-FAPI-04 PET/CT Images of Lung Cancer Patients

Upon visual inspection of the ^68^Ga-FAPI-04 PET/CT images, all pathologically confirmed lung cancer could be seen at the first phase. However, the images acquired in the late phase offered greater visualization (contrast) of the positive ^68^Ga-FAPI-04 uptake in all tumors than those acquired in the early phase (Figures 1A,B).

**Figure 5:**
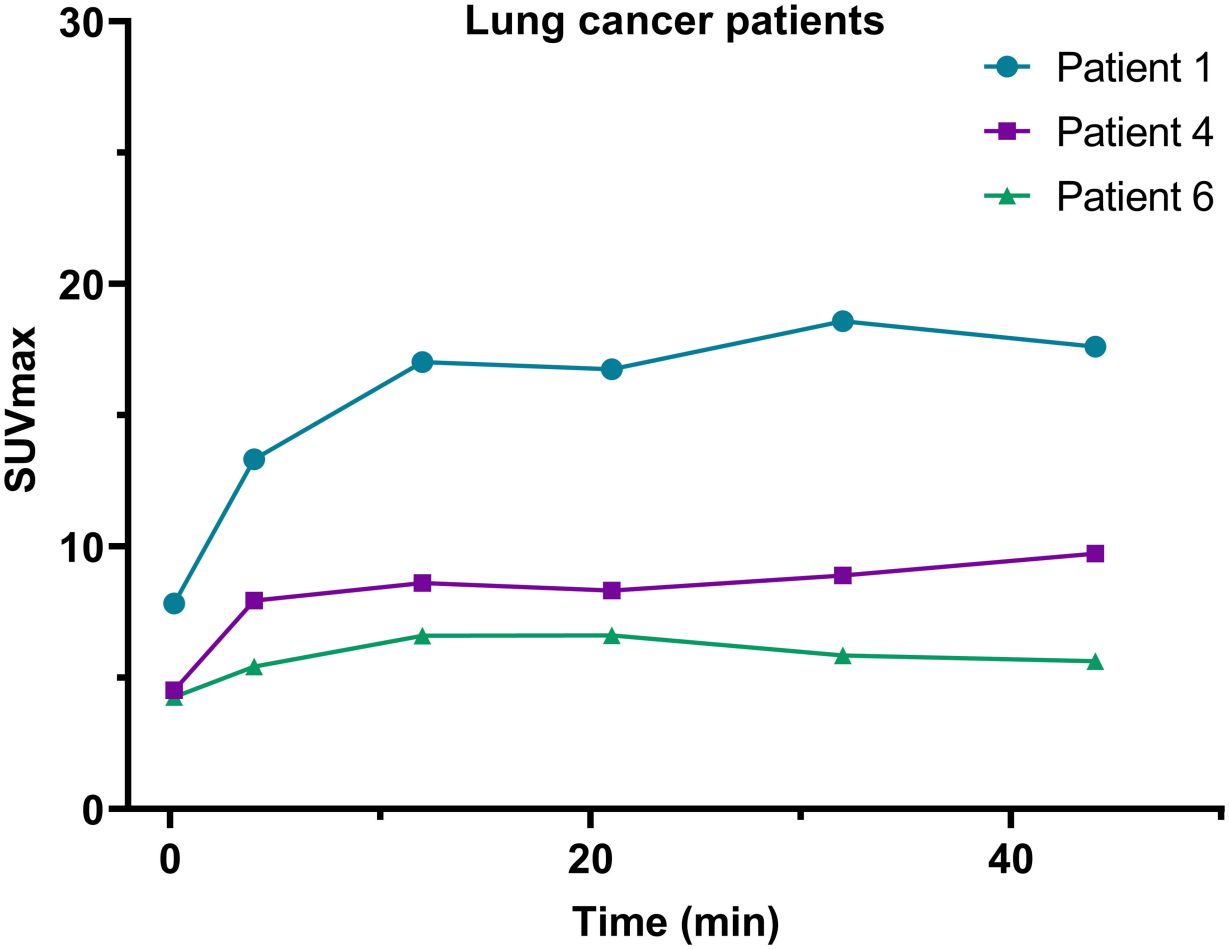
Variations in SUVmax on ^68^Ga-FAPI-04 PET/CT images of the 3 lung cancer patients included in this study.

Quantitatively, in this dynamic study, we found that the SUVpeak of ^68^Ga-FAPI-04 in the lung tumors was apparent in the late phases. The SURs (tumor uptake to background ratios) of lung cancer (SUR_4min_ = 5.5; SUR_12min_ = 8.2; SUR_21min_ = 8.5; SUR_44min_ = 11.7) increased from the early-phase acquisitions to the late-phase acquisitions. Variations in ^68^Ga-FAPI-04 uptake (SUVmax) in the lesions of lung cancer patients are presented in Figure 5, and the SUVmax and SUR values of lung cancer patients at different time points are shown in Supplementary Table 5.

**Figure 6:**
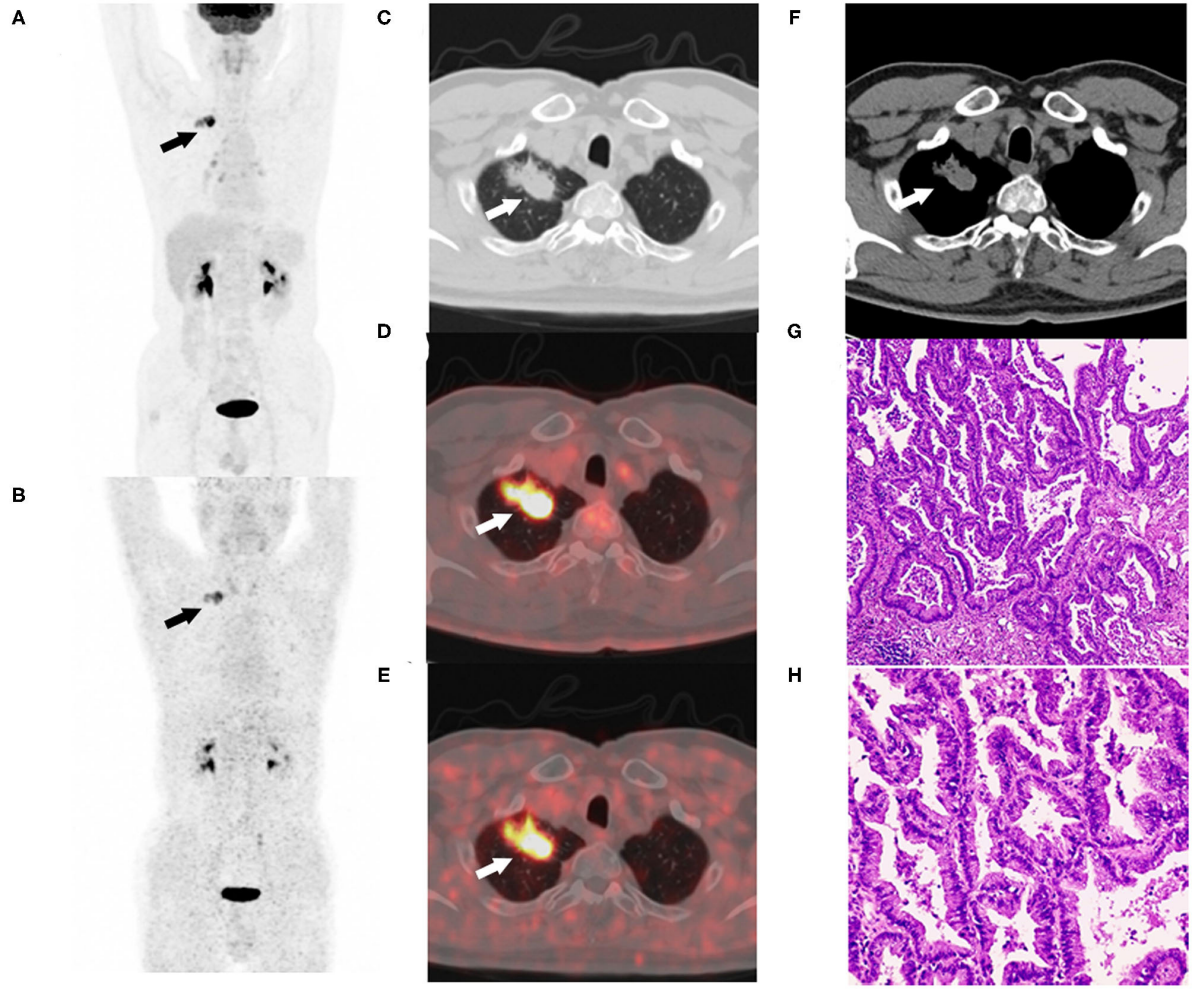
Sixty-two-year-old male with adenocarcinoma of the right upper lung (patient 4). Maximum intensity projection of 2-[^18^F]FDG **(A)** and ^68^Ga-FAPI-04 PET/CT examinations **(B)**. The lobulated and spiculated nodule of the right upper lung were observed in CT images **(C,F)**. The nodule exhibited high uptake of 2-[^18^F]FDG (SUVmax = 16) **(D)** and high uptake of ^68^Ga-FAPI-04 (SUVmax = 9.8) **(E)**. The resected specimens were confirmed as adenocarcinoma by hematoxylin-eosin staining **(G)** ×40, **(H)** ×100.

The patients involved in this study demonstrated representative ^68^Ga-FAPI-04 uptake in lung cancer. The 62-year-old male patient with right lung cancer underwent paired 2-[^18^F]FDG and ^68^Ga-FAPI-04 PET/CT examinations. The tumor exhibited high uptake of 2-[^18^F]FDG (SUVmax = 16) and high uptake of ^68^Ga-FAPI-04 (SUVmax = 9.8). The tumor was confirmed as adenocarcinoma by resected specimen (Figure 6), demonstrating the good imaging efficacy of ^68^Ga-FAPI-04 for lung cancer.

## Discussion

Fibroblast activation protein (FAP) is closely associated with the invasion and metastasis of human cancers and has been revealed as a promising theranostic target for tumors. In the past several years, many small-molecule FAPIs based on different chemical structures have been fabricated, and their utility as a tumor theranostic has been evaluated. To further investigate the biodistribution, tumor uptake and retention of ^68^Ga-FAPI-04, the results of a series of dynamic PET/CT imaging studies of both healthy subjects and oncological patients after an intravenous injection of ^68^Ga-FAPI-04 were reported and analyzed.

In this work, similar to other clinical trials with FAPIs (16–18), the ^68^Ga-FAPI-04 probe was cleared rapidly from the human body through renal excretion, rendering low background uptake in most normal organs (Table 2). This clearance was especially noticeable in the lung (SUVmean = 0.4 ± 0.1), liver (SUVmean = 0.6 ± 0.1), and brain (SUVmean = 0.02 ± 0.03) at the later time points, indicating that ^68^Ga-FPAI-04 is quite suitable for the imaging of tumor lesions in these organs. Regarding the *in vivo* biodistribution of ^68^Ga-FAPI-04 in most organs, there was no significant difference between male and female patients. We found that the SUVmean of the thyroid of Chinese male patients was higher than that in female patients at time point 1 (9.0 ± 0.5 vs. 4.8 ± 2.0, *p* = 0.05), while there was no difference at later time points (2.4 ± 1.1 vs. 2.1 ± 1.1, not statistically significant). This might be because of individual differences, since the CV of the SUVmean of the thyroid of female subjects at time point 1 was 40.7%, while the CV of the SUVmean of the thyroid of male subjects was only 5.6%. Similar to a previous study using ^68^Ga-FAPI-46 as an imaging agent for cancer patients, local uptake in the uterus of female subjects increased slowly within 1 h postinjection, which might be because of the higher physiological expression of FAP in the uterus (16). Since none of the patients drank water or were administered furosemide after the injection, the clearance of the ^68^Ga-FAPI-04 probe from the muscle would be slower in these patients than in patients administered furosemide in a previous study (SUVmean = 1.0, SUVmax = 1.4) (12), leading to higher uptake (SUVmean = 1.3 and SUVmax = 2.0) in the muscle at later time points.

Furthermore, the SUL has been preferred in the evaluation of biodistribution of ^18^F-radiolabeled probes, since the SUL was not correlated with body mass (19). In this study, we also compared the SUVmean with the SULmean in most organs across all patients, as shown in Tables 2, 3. At approximately 40 min postinjection, we found that the SUVmean was slightly higher than the SULmean in most organs, with no significant difference; however, the CV of the SULmean was higher than that of the SUVmean in most organs. Although the SULmean of more patients was within the 95% confidence interval with respect to the lung and liver, as shown in Figure 4, the SUVmean was applied in our study to evaluate the biodistribution of ^68^Ga-FAPI-04 in normal organs. The variations in the uptake in normal organs on ^68^Ga-FAPI-04 PET scans, as were measured by the CV, need to be understood to allow for uptake changes in malignant lesions to be confidently attributed to changes in disease or treatment response rather than inherent variability among scans. For these reasons, we analyzed the quantitative aspects of ^68^Ga-FAPI-04 imaging as they relate to normal organ variability. The liver has a moderate level of 2-[^18^F]FDG uptake in the normal parenchyma and a low uptake variability relative to other organs. This organ was chosen for further evaluations of quantitative reliability among PET images (20). In this study, liver uptake showed a low variability (CV = 12.4%) when using SUVmean, which was similar to the results of 2-[^18^F]FDG (11.8%). This finding implies that ^68^Ga-FAPI-04 PET images can be reliably quantified, laying the groundwork for future studies involving therapeutic monitoring.

In our study, the absorbed dose of all organs was determined based on dynamic PET/CT imaging. During the whole imaging period, no patients voided their bladders, thus causing the radioactivity in the bladder to increase continuously. Hence, biexponential curve fitting was applied to the activity concentration-time curves of urinary bladder content. Similar to the dosimetry results in other studies on ^68^Ga radiolabeled probes, the urinary bladder had the highest absorbed dose as well as the highest effective dose. Interestingly, we noticed that, other than the urinary bladder wall, among all other normal organs, the lung had the highest organ effective dose (1.92E-03 mSv/MBq), which might be because the three subjects included in this study were lung cancer patients. The absorbed doses of most organs, except the uterus and the whole-body effective dose of ^68^Ga-FAPI-04 PET imaging in our study (1.26E-02 mSv/MBq), were lower than the previously reported results (1.46E-02 mSv/MBq) in Caucasian patients (11), which might be because of individual differences between these two cohorts. An administration of 100 MBq of ^68^Ga-FAPI-04 would result in only a 1.26 mSv effective dose for adult patients, which is much lower than the 3.7 mSv of a whole-body scan and 1.8 mSv of a scan of the abdomen at 1 bed position with low-dose CT in adults (15, 21). Such a low effective dose would allow patients to receive 2–4 repeated ^68^Ga-FAPI-04 PET/CT imaging procedures per year and up to 8 PET/MRI examinations without exceeding the maximum dose of 10 mSv (21). Even so, the kidney absorbed dose would remain well below the threshold for the human kidney acute dose (7 Gy) (22). Furthermore, the absorbed dose in the red marrow was only 0.65 mGy/100 MBq, and even with up to 380 examinations, the absorbed dose in the red marrow would still be <0.25 Gy per year, which would not result in any damaging effects (22).

According to the statistics from OLINDA/EXM 2.0, body remainder had the longest residence time (0.584 h), followed by the urinary bladder (0.102 h), lungs (0.037 h), liver (0.018 h) and kidneys (0.013 h). Compared with a previously reported study of a ^68^Ga radiolabeled probe, the residence times in this study for most organs were shorter than the values reported for the ^68^Ga-DOTA^ZOL^ probe, especially for the liver (0.018 vs. 0.040) and spleen (0.003 vs. 0.005), indicating that ^68^Ga-FAPI-04 was quite stable in the human body, with less free/unbound ^68^Ga than the ^68^Ga-DOTA^ZOL^ probe (23). However, all these results might be limited by the subjects included in our study, since there were only 3 cancer patients and 3 healthy volunteers in this study. Furthermore, the expression of FAP due to wound healing, arthritis, inflammation and cirrhotic liver would interfere with the results (24–26).

In addition, in this study, we found that the SUVmax in lesions of lung cancer patients increased during this dynamic scan, and the background organ/tissue ratio decreased over time. Therefore, these results suggest that late imaging should be used for lung cancer patients, although the significance of ^68^Ga-FAPI-04 imaging for lung cancer needs to be investigated with more subjects.

## Conclusion

The first biodistribution and internal radiation dosimetry profiles in the adult Chinese population following the administration of a ^68^Ga-FAPI-04 injection have been presented. A comparison of these data with those obtained before in the Caucasian population demonstrated no clinically significant differences between these two populations after ^68^Ga-FAPI-04 injections. In particular, the variability of ^68^Ga-FAPI-04 uptake in normal lungs was lower than that of 2-[^18^F]FDG. This finding implies that ^68^Ga-FAPI-04 PET images of lung cancer patients can be reliably quantified, thus laying the groundwork for future studies involving therapeutic monitoring. Moreover, a high SUR can be achieved as early as 12 min postinjection of ^68^Ga-FAPI-04, indicating that high-quality diagnostic images of lung cancer patients can be acquired at earlier time points than with 2-[^18^F]FDG. Furthermore, the variability in SUVmean for most organs was marginally lower than that in SULmean, favoring the adoption of SUVmean for ^68^Ga-FAPI-04 PET scans. In addition, ^68^Ga-FAPI-04 PET/CT imaging is expected to be a promising tool for diagnosing lung cancer.

## Data Availability Statement

The original contributions presented in the study are included in the article/Supplementary Material, further inquiries can be directed to the corresponding author/s.

## Ethics Statement

The studies involving human participants were reviewed and approved by Ethics Committee of Peking University Cancer Hospital. The patients/participants provided their written informed consent to participate in this study. The animal study was reviewed and approved by Peking University Cancer Hospital Animal Care and Use Committee.

## Author Contributions

ZY, HZ, and NL conceived and designed this research. SW, XZ, and XX were responsible to the recruitment of patients, conducting of clinical patients experiments, data collection and analysis, and they also wrote the manuscript. JD and TL were involvode in the preparation of radiopharmaceuticals in this study. JJ took part in most of the animal experiments. All of the authors joined in the embellishment of the article.

## Conflict of Interest

The authors declare that the research was conducted in the absence of any commercial or financial relationships that could be construed as a potential conflict of interest.
