# Supplementary material for: Dynamic PET/CT Imaging of 68Ga-FAPI-04 in Chinese Subjects
Source: Front Oncol. 2021 Mar 11;11:651005. doi: 10.3389/fonc.2021.651005 (PMC7991833; doi:10.3389/fonc.2021.651005)
Supplement: Supplementary file 1 [file Data_Sheet_1.docx]

Supplementary Material

## Supplementary Figures

**
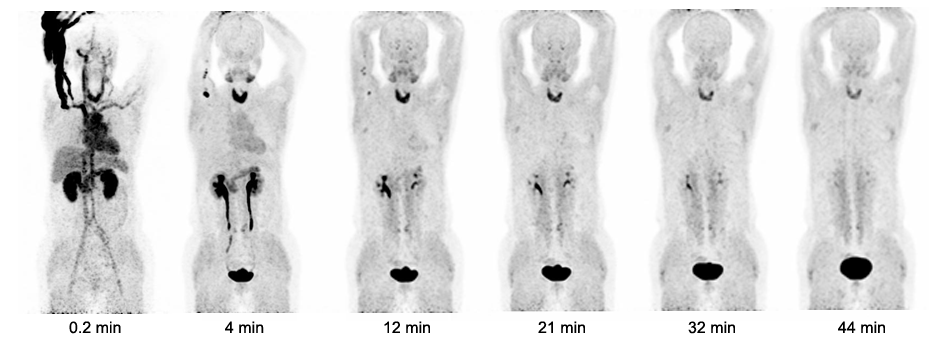
**

**Supplementary Figure 1.** PET MIP images of female healthy volunteer 2.

**Dynamic PET/CT imaging and biodistribution of ^68^Ga-FAPI-04 in KM mice**

In the animal microPET imaging experiment, KM mice (4 weeks, female, 18-20 g) were anesthetized with isoflurane and placed on the imaging bed, venous access through the tail vein was further established. At the same time as the injection of 7.4 MBq of ^68^Ga-FAPI-04, the PET imaging was started and continued for 90 minutes with Mira® small animal PET/CT (PINGSENG Healthcare, China).

In the animal biodistribution experiment, 1.85 MBq of ^68^Ga-FAPI-04 was administered intravenously to KM mice (4 weeks, female, 18-20 g). Animals were sacrificed at 5 min, 10 min, 20 min, 30 min, 1 h and 2 h post injection (n = 3), organs of interests were resected, weighted and counted using a gamma counter (Perkin Elmer, Waltham, MA, USA). The biodistribution data (% ID/g) were expressed as the mean ± standard deviation. All animal studies were performed according to a protocol approved by the Peking University Cancer Hospital Animal Care and Use Committee.


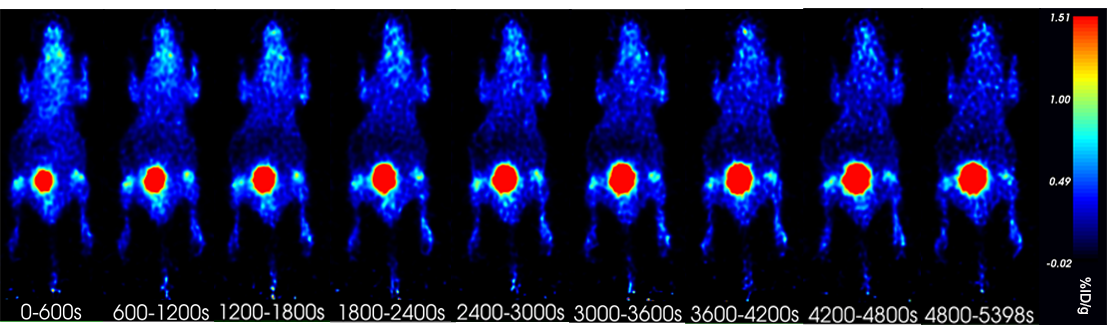


**Supplementary Figure 2.** Dynamic micro PET/CT imaging of ^68^Ga-FAPI-04 in KM mouse lasted for 90 minutes.


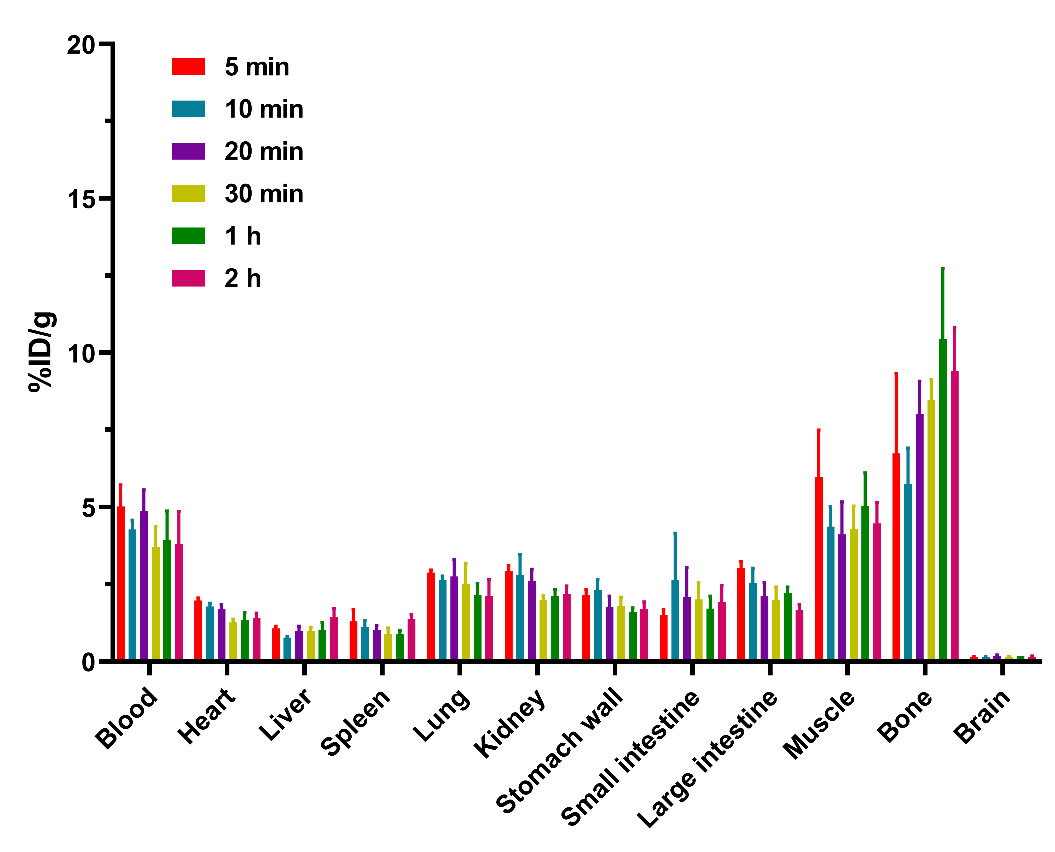


**Supplementary Figure 3.** Biodistrubution analysis of ^68^Ga-FAPI-04 in KM mice at different time points.

## Supplementary Tables

**Supplementary Table 1.** Dosimetry results of each patient calculated from OLINDA/EXM 2.0.

**Patient 1**

| Target Organ | Alpha | Beta | Gamma | Total | ICRP-103 ED |
| --- | --- | --- | --- | --- | --- |
| Adrenals | 0.00E+00 | 5.81E-03 | 3.51E-03 | 9.32E-03 | 8.61E-05 |
| Brain | 0.00E+00 | 4.87E-03 | 2.06E-03 | 6.92E-03 | 6.92E-05 |
| Breasts | 0.00E+00 | 4.87E-03 | 1.95E-03 | 6.81E-03 | 8.18E-04 |
| Esophagus | 0.00E+00 | 4.87E-03 | 2.70E-03 | 7.57E-03 | 3.03E-04 |
| Eyes | 0.00E+00 | 4.87E-03 | 2.06E-03 | 6.92E-03 | 0.00E+00 |
| Gallbladder Wall | 0.00E+00 | 4.87E-03 | 3.20E-03 | 8.07E-03 | 7.45E-05 |
| Left colon | 0.00E+00 | 4.87E-03 | 3.45E-03 | 8.32E-03 | 4.03E-04 |
| Small Intestine | 0.00E+00 | 4.90E-03 | 3.24E-03 | 8.14E-03 | 7.51E-05 |
| Stomach Wall | 0.00E+00 | 4.98E-03 | 3.29E-03 | 8.27E-03 | 9.93E-04 |
| Right colon | 0.00E+00 | 4.88E-03 | 3.31E-03 | 8.19E-03 | 3.97E-04 |
| Rectum | 0.00E+00 | 5.14E-03 | 5.18E-03 | 1.03E-02 | 2.37E-04 |
| Heart Wall | 0.00E+00 | 8.75E-03 | 3.57E-03 | 1.23E-02 | 1.14E-04 |
| Kidneys | 0.00E+00 | 1.18E-02 | 3.62E-03 | 1.54E-02 | 1.43E-04 |
| Liver | 0.00E+00 | 4.17E-03 | 2.93E-03 | 7.10E-03 | 2.84E-04 |
| Lungs | 0.00E+00 | 1.31E-02 | 3.16E-03 | 1.63E-02 | 1.95E-03 |
| Ovaries | 0.00E+00 | 4.87E-03 | 4.24E-03 | 9.12E-03 | 3.65E-04 |
| Pancreas | 0.00E+00 | 1.44E-02 | 4.28E-03 | 1.87E-02 | 1.73E-04 |
| Salivary Glands | 0.00E+00 | 4.87E-03 | 2.31E-03 | 7.18E-03 | 7.18E-05 |
| Red Marrow | 0.00E+00 | 5.13E-03 | 2.96E-03 | 8.09E-03 | 9.70E-04 |
| Osteogenic Cells | 0.00E+00 | 3.06E-03 | 2.96E-03 | 6.03E-03 | 6.03E-05 |
| Spleen | 0.00E+00 | 7.23E-03 | 3.36E-03 | 1.06E-02 | 9.78E-05 |
| Thymus | 0.00E+00 | 5.41E-03 | 3.14E-03 | 8.55E-03 | 7.89E-05 |
| Thyroid | 0.00E+00 | 4.87E-03 | 2.52E-03 | 7.38E-03 | 2.95E-04 |
| UB Wall | 0.00E+00 | 5.30E-02 | 6.69E-03 | 5.97E-02 | 2.39E-03 |
| Uterus | 0.00E+00 | 2.30E-02 | 6.46E-03 | 2.94E-02 | 1.36E-04 |
| Total Body | 0.00E+00 | 5.69E-03 | 2.57E-03 | 8.26E-03 | 0.00E+00 |
| Effective Dose (mSv/MBq) | |  |  | 1.06E-02 | |

**Patient 2**

| Target Organ | Alpha | Beta | Gamma | Total | ICRP-103 ED |
| --- | --- | --- | --- | --- | --- |
| Adrenals | 0.00E+00 | 5.71E-03 | 3.37E-03 | 9.09E-03 | 8.39E-05 |
| Brain | 0.00E+00 | 4.87E-03 | 2.04E-03 | 6.90E-03 | 6.90E-05 |
| Breasts | 0.00E+00 | 4.87E-03 | 1.90E-03 | 6.76E-03 | 8.12E-04 |
| Esophagus | 0.00E+00 | 4.87E-03 | 2.53E-03 | 7.40E-03 | 2.96E-04 |
| Eyes | 0.00E+00 | 4.87E-03 | 2.04E-03 | 6.90E-03 | 0.00E+00 |
| Gallbladder Wall | 0.00E+00 | 4.87E-03 | 3.15E-03 | 8.02E-03 | 7.40E-05 |
| Left colon | 0.00E+00 | 4.87E-03 | 3.41E-03 | 8.28E-03 | 4.01E-04 |
| Small Intestine | 0.00E+00 | 4.88E-03 | 3.22E-03 | 8.10E-03 | 7.48E-05 |
| Stomach Wall | 0.00E+00 | 4.95E-03 | 3.11E-03 | 8.06E-03 | 9.67E-04 |
| Right colon | 0.00E+00 | 4.88E-03 | 3.29E-03 | 8.18E-03 | 3.97E-04 |
| Rectum | 0.00E+00 | 5.01E-03 | 5.64E-03 | 1.06E-02 | 2.45E-04 |
| Heart Wall | 0.00E+00 | 7.80E-03 | 3.28E-03 | 1.11E-02 | 1.02E-04 |
| Kidneys | 0.00E+00 | 1.57E-02 | 3.90E-03 | 1.96E-02 | 1.81E-04 |
| Liver | 0.00E+00 | 3.76E-03 | 2.78E-03 | 6.54E-03 | 2.62E-04 |
| Lungs | 0.00E+00 | 1.15E-02 | 2.98E-03 | 1.44E-02 | 1.73E-03 |
| Ovaries | 0.00E+00 | 4.87E-03 | 4.33E-03 | 9.20E-03 | 3.68E-04 |
| Pancreas | 0.00E+00 | 3.70E-03 | 3.25E-03 | 6.95E-03 | 6.42E-05 |
| Salivary Glands | 0.00E+00 | 4.87E-03 | 2.29E-03 | 7.16E-03 | 7.16E-05 |
| Red Marrow | 0.00E+00 | 4.23E-03 | 2.90E-03 | 7.13E-03 | 8.56E-04 |
| Osteogenic Cells | 0.00E+00 | 2.61E-03 | 2.92E-03 | 5.53E-03 | 5.53E-05 |
| Spleen | 0.00E+00 | 4.89E-03 | 3.12E-03 | 8.01E-03 | 7.39E-05 |
| Thymus | 0.00E+00 | 5.34E-03 | 2.99E-03 | 8.33E-03 | 7.68E-05 |
| Thyroid | 0.00E+00 | 4.87E-03 | 2.45E-03 | 7.31E-03 | 2.93E-04 |
| UB Wall | 0.00E+00 | 7.08E-02 | 8.18E-03 | 7.90E-02 | 3.16E-03 |
| Uterus | 0.00E+00 | 1.20E-02 | 6.20E-03 | 1.82E-02 | 8.43E-05 |
| Total Body | 0.00E+00 | 5.68E-03 | 2.56E-03 | 8.24E-03 | 0.00E+00 |
| Effective Dose (mSv/MBq) | |  |  | 1.08E-02 | |

**Patient 3**

| Target Organ | Alpha | Beta | Gamma | Total | ICRP-103 ED |
| --- | --- | --- | --- | --- | --- |
| Adrenals | 0.00E+00 | 4.22E-03 | 3.64E-03 | 7.85E-03 | 7.25E-05 |
| Brain | 0.00E+00 | 2.40E-03 | 1.05E-03 | 3.45E-03 | 3.45E-05 |
| Breasts | 0.00E+00 | 2.40E-03 | 1.21E-03 | 3.61E-03 | 4.34E-04 |
| Esophagus | 0.00E+00 | 2.41E-03 | 2.18E-03 | 4.59E-03 | 1.84E-04 |
| Eyes | 0.00E+00 | 2.40E-03 | 1.05E-03 | 3.46E-03 | 0.00E+00 |
| Gallbladder Wall | 0.00E+00 | 2.41E-03 | 3.05E-03 | 5.46E-03 | 5.04E-05 |
| Left colon | 0.00E+00 | 2.41E-03 | 3.54E-03 | 5.95E-03 | 2.88E-04 |
| Small Intestine | 0.00E+00 | 2.44E-03 | 4.40E-03 | 6.84E-03 | 6.32E-05 |
| Stomach Wall | 0.00E+00 | 2.54E-03 | 2.38E-03 | 4.92E-03 | 5.90E-04 |
| Right colon | 0.00E+00 | 2.44E-03 | 2.72E-03 | 5.16E-03 | 2.50E-04 |
| Rectum | 0.00E+00 | 3.00E-03 | 1.95E-02 | 2.25E-02 | 5.18E-04 |
| Heart Wall | 0.00E+00 | 6.19E-03 | 2.73E-03 | 8.91E-03 | 8.23E-05 |
| Kidneys | 0.00E+00 | 2.89E-02 | 4.95E-03 | 3.38E-02 | 3.12E-04 |
| Liver | 0.00E+00 | 8.85E-03 | 3.42E-03 | 1.23E-02 | 4.91E-04 |
| Lungs | 0.00E+00 | 1.45E-02 | 2.45E-03 | 1.70E-02 | 2.04E-03 |
| Ovaries | 0.00E+00 | 2.42E-03 | 9.85E-03 | 1.23E-02 | 4.91E-04 |
| Pancreas | 0.00E+00 | 6.45E-03 | 3.19E-03 | 9.64E-03 | 8.90E-05 |
| Salivary Glands | 0.00E+00 | 2.40E-03 | 1.21E-03 | 3.62E-03 | 3.62E-05 |
| Red Marrow | 0.00E+00 | 3.12E-03 | 3.11E-03 | 6.23E-03 | 7.47E-04 |
| Osteogenic Cells | 0.00E+00 | 1.81E-03 | 2.25E-03 | 4.06E-03 | 4.06E-05 |
| Spleen | 0.00E+00 | 1.15E-02 | 3.24E-03 | 1.47E-02 | 1.36E-04 |
| Thymus | 0.00E+00 | 3.00E-03 | 2.17E-03 | 5.17E-03 | 4.77E-05 |
| Thyroid | 0.00E+00 | 2.41E-03 | 1.55E-03 | 3.96E-03 | 1.58E-04 |
| UB Wall | 0.00E+00 | 4.57E-01 | 4.29E-02 | 5.00E-01 | 2.00E-02 |
| Uterus | 0.00E+00 | 5.04E-02 | 2.31E-02 | 7.34E-02 | 3.39E-04 |
| Total Body | 0.00E+00 | 5.64E-03 | 2.67E-03 | 8.31E-03 | 0.00E+00 |
| Effective Dose (mSv/MBq) | |  |  | 2.75E-02 | |

**Patient 4**

| Target Organ | Alpha | | Beta | Gamma | Total | ICRP-103 ED |
| --- | --- | --- | --- | --- | --- | --- |
| Adrenals | 0.00E+00 | | 4.48E-03 | 3.07E-03 | 7.55E-03 | 6.97E-05 |
| Brain | 0.00E+00 | | 3.72E-03 | 1.55E-03 | 5.27E-03 | 5.27E-05 |
| Esophagus | 0.00E+00 | | 3.72E-03 | 2.89E-03 | 6.61E-03 | 2.64E-04 |
| Eyes | 0.00E+00 | | 3.72E-03 | 1.55E-03 | 5.27E-03 | 0.00E+00 |
| Gallbladder Wall | 0.00E+00 | | 3.86E-03 | 2.78E-03 | 6.65E-03 | 6.14E-05 |
| Left colon | 0.00E+00 | | 3.75E-03 | 2.79E-03 | 6.54E-03 | 3.17E-04 |
| Small Intestine | 0.00E+00 | | 3.74E-03 | 2.85E-03 | 6.59E-03 | 6.08E-05 |
| Stomach Wall | 0.00E+00 | | 3.95E-03 | 3.04E-03 | 6.98E-03 | 8.38E-04 |
| Right colon | 0.00E+00 | | 3.72E-03 | 2.66E-03 | 6.38E-03 | 3.09E-04 |
| Rectum | 0.00E+00 | | 3.72E-03 | 3.26E-03 | 6.97E-03 | 1.60E-04 |
| Heart Wall | 0.00E+00 | | 8.69E-03 | 3.53E-03 | 1.22E-02 | 1.13E-04 |
| Kidneys | 0.00E+00 | | 1.27E-02 | 3.31E-03 | 1.60E-02 | 1.48E-04 |
| Liver | 0.00E+00 | | 4.07E-03 | 2.65E-03 | 6.72E-03 | 2.69E-04 |
| Lungs | 0.00E+00 | | 1.95E-02 | 3.20E-03 | 2.27E-02 | 2.72E-03 |
| Pancreas | 0.00E+00 | | 1.13E-02 | 3.62E-03 | 1.49E-02 | 1.38E-04 |
| Prostate | 0.00E+00 | | 3.72E-03 | 3.64E-03 | 7.36E-03 | 3.40E-05 |
| Salivary Glands | 0.00E+00 | | 3.72E-03 | 2.01E-03 | 5.72E-03 | 5.72E-05 |
| Red Marrow | 0.00E+00 | | 3.49E-03 | 2.42E-03 | 5.90E-03 | 7.08E-04 |
| Osteogenic Cells | 0.00E+00 | | 2.78E-03 | 2.38E-03 | 5.16E-03 | 5.16E-05 |
| Spleen | 0.00E+00 | | 1.26E-02 | 3.38E-03 | 1.60E-02 | 1.48E-04 |
| Testes | 0.00E+00 | | 3.72E-03 | 2.09E-03 | 5.81E-03 | 2.33E-04 |
| Thymus | 0.00E+00 | | 3.72E-03 | 2.80E-03 | 6.52E-03 | 6.02E-05 |
| Thyroid | 0.00E+00 | | 3.72E-03 | 2.49E-03 | 6.21E-03 | 2.48E-04 |
| UB Wall | 0.00E+00 | | 4.60E-02 | 8.33E-03 | 5.43E-02 | 2.17E-03 |
| Total Body | 0.00E+00 | | 4.62E-03 | 2.00E-03 | 6.61E-03 | 0.00E+00 |
| Effective Dose (mSv/MBq) | |  | |  | 9.23E-03 | |

**Patient 5**

| Target Organ | Alpha | | Beta | Gamma | Total | ICRP-103 ED |
| --- | --- | --- | --- | --- | --- | --- |
| Adrenals | 0.00E+00 | | 4.76E-03 | 3.13E-03 | 7.89E-03 | 7.29E-05 |
| Brain | 0.00E+00 | | 3.68E-03 | 1.52E-03 | 5.19E-03 | 5.19E-05 |
| Esophagus | 0.00E+00 | | 3.68E-03 | 2.34E-03 | 6.02E-03 | 2.41E-04 |
| Eyes | 0.00E+00 | | 3.68E-03 | 1.52E-03 | 5.20E-03 | 0.00E+00 |
| Gallbladder Wall | 0.00E+00 | | 3.81E-03 | 2.67E-03 | 6.48E-03 | 5.98E-05 |
| Left colon | 0.00E+00 | | 3.70E-03 | 2.78E-03 | 6.47E-03 | 3.14E-04 |
| Small Intestine | 0.00E+00 | | 3.69E-03 | 3.12E-03 | 6.80E-03 | 6.28E-05 |
| Stomach Wall | 0.00E+00 | | 3.79E-03 | 2.59E-03 | 6.38E-03 | 7.66E-04 |
| Right colon | 0.00E+00 | | 3.68E-03 | 2.73E-03 | 6.41E-03 | 3.11E-04 |
| Rectum | 0.00E+00 | | 3.68E-03 | 4.50E-03 | 8.18E-03 | 1.88E-04 |
| Heart Wall | 0.00E+00 | | 8.22E-03 | 2.96E-03 | 1.12E-02 | 1.03E-04 |
| Kidneys | 0.00E+00 | | 1.94E-02 | 3.94E-03 | 2.33E-02 | 2.15E-04 |
| Liver | 0.00E+00 | | 3.74E-03 | 2.39E-03 | 6.13E-03 | 2.45E-04 |
| Lungs | 0.00E+00 | | 8.73E-03 | 2.31E-03 | 1.10E-02 | 1.32E-03 |
| Pancreas | 0.00E+00 | | 6.02E-03 | 2.95E-03 | 8.97E-03 | 8.28E-05 |
| Prostate | 0.00E+00 | | 3.68E-03 | 5.48E-03 | 9.16E-03 | 4.23E-05 |
| Salivary Glands | 0.00E+00 | | 3.68E-03 | 1.94E-03 | 5.62E-03 | 5.62E-05 |
| Red Marrow | 0.00E+00 | | 3.24E-03 | 2.42E-03 | 5.67E-03 | 6.80E-04 |
| Osteogenic Cells | 0.00E+00 | | 2.62E-03 | 2.38E-03 | 5.00E-03 | 5.00E-05 |
| Spleen | 0.00E+00 | | 5.47E-03 | 2.64E-03 | 8.11E-03 | 7.48E-05 |
| Testes | 0.00E+00 | | 3.68E-03 | 2.51E-03 | 6.19E-03 | 2.48E-04 |
| Thymus | 0.00E+00 | | 3.68E-03 | 2.33E-03 | 6.01E-03 | 5.54E-05 |
| Thyroid | 0.00E+00 | | 3.68E-03 | 2.11E-03 | 5.79E-03 | 2.32E-04 |
| UB Wall | 0.00E+00 | | 1.07E-01 | 1.73E-02 | 1.24E-01 | 4.95E-03 |
| Total Body | 0.00E+00 | | 4.72E-03 | 2.07E-03 | 6.79E-03 | 0.00E+00 |
| Effective Dose (mSv/MBq) | |  | |  | 1.04E-02 | |

**Patient 6**

| Target Organ | Alpha | Beta | Gamma | Total | ICRP-103 ED |
| --- | --- | --- | --- | --- | --- |
| Adrenals | 0.00E+00 | 4.08E-03 | 2.91E-03 | 7.00E-03 | 6.46E-05 |
| Brain | 0.00E+00 | 3.05E-03 | 1.27E-03 | 4.31E-03 | 4.31E-05 |
| Esophagus | 0.00E+00 | 3.05E-03 | 2.37E-03 | 5.42E-03 | 2.17E-04 |
| Eyes | 0.00E+00 | 3.05E-03 | 1.27E-03 | 4.32E-03 | 0.00E+00 |
| Gallbladder Wall | 0.00E+00 | 3.20E-03 | 2.44E-03 | 5.64E-03 | 5.20E-05 |
| Left colon | 0.00E+00 | 3.07E-03 | 2.33E-03 | 5.40E-03 | 2.62E-04 |
| Small Intestine | 0.00E+00 | 3.06E-03 | 2.40E-03 | 5.45E-03 | 5.03E-05 |
| Stomach Wall | 0.00E+00 | 3.19E-03 | 2.49E-03 | 5.68E-03 | 6.82E-04 |
| Right colon | 0.00E+00 | 3.05E-03 | 2.25E-03 | 5.30E-03 | 2.57E-04 |
| Rectum | 0.00E+00 | 3.05E-03 | 2.80E-03 | 5.85E-03 | 1.35E-04 |
| Heart Wall | 0.00E+00 | 9.84E-03 | 3.35E-03 | 1.32E-02 | 1.22E-04 |
| Kidneys | 0.00E+00 | 1.81E-02 | 3.56E-03 | 2.16E-02 | 2.00E-04 |
| Liver | 0.00E+00 | 4.22E-03 | 2.40E-03 | 6.62E-03 | 2.65E-04 |
| Lungs | 0.00E+00 | 1.22E-02 | 2.44E-03 | 1.46E-02 | 1.76E-03 |
| Pancreas | 0.00E+00 | 6.22E-03 | 2.77E-03 | 9.00E-03 | 8.30E-05 |
| Prostate | 0.00E+00 | 3.05E-03 | 3.18E-03 | 6.23E-03 | 2.88E-05 |
| Salivary Glands | 0.00E+00 | 3.05E-03 | 1.63E-03 | 4.68E-03 | 4.68E-05 |
| Red Marrow | 0.00E+00 | 2.69E-03 | 1.98E-03 | 4.67E-03 | 5.60E-04 |
| Osteogenic Cells | 0.00E+00 | 2.17E-03 | 1.96E-03 | 4.13E-03 | 4.13E-05 |
| Spleen | 0.00E+00 | 1.04E-02 | 2.87E-03 | 1.33E-02 | 1.23E-04 |
| Testes | 0.00E+00 | 3.05E-03 | 1.76E-03 | 4.81E-03 | 1.92E-04 |
| Thymus | 0.00E+00 | 3.05E-03 | 2.37E-03 | 5.42E-03 | 5.01E-05 |
| Thyroid | 0.00E+00 | 3.05E-03 | 1.95E-03 | 5.00E-03 | 2.00E-04 |
| UB Wall | 0.00E+00 | 4.35E-02 | 7.69E-03 | 5.12E-02 | 2.05E-03 |
| Total Body | 0.00E+00 | 3.83E-03 | 1.65E-03 | 5.49E-03 | 0.00E+00 |
| Effective Dose (mSv/MBq) | |  |  | 7.48E-03 | |

**Supplementary Table 2.** Average organ effective doses (mSv/MBq) across all patients acquired using reference adult models in OLINDA/EXM 2.0.

| Target Organ | Mean | SD |
| --- | --- | --- |
| Adrenals | 7.50E-05 | 8.36E-06 |
| Brain | 5.34E-05 | 1.38E-05 |
| Breasts | 6.88E-04 | 2.20E-04 |
| Esophagus | 2.51E-04 | 4.61E-05 |
| Eyes | 0.00E+00 | 0.00E+00 |
| Gallbladder Wall | 6.20E-05 | 1.04E-05 |
| Left colon | 3.31E-04 | 5.86E-05 |
| Small Intestine | 6.45E-05 | 9.36E-06 |
| Stomach Wall | 8.06E-04 | 1.58E-04 |
| Right colon | 3.20E-04 | 6.47E-05 |
| Rectum | 2.47E-04 | 1.39E-04 |
| Heart Wall | 1.06E-04 | 1.38E-05 |
| Kidneys | 2.00E-04 | 6.18E-05 |
| Liver | 3.03E-04 | 9.31E-05 |
| Lungs | 1.92E-03 | 4.64E-04 |
| Ovaries | 4.08E-04 | 7.19E-05 |
| Pancreas | 1.05E-04 | 4.15E-05 |
| Prostate | 3.50E-05 | 6.81E-06 |
| Salivary Glands | 5.66E-05 | 1.39E-05 |
| Red Marrow | 7.54E-04 | 1.43E-04 |
| Osteogenic Cells | 4.99E-05 | 7.75E-06 |
| Spleen | 1.09E-04 | 3.15E-05 |
| Testes | 2.24E-04 | 2.90E-05 |
| Thymus | 6.15E-05 | 1.34E-05 |
| Thyroid | 2.38E-04 | 5.34E-05 |
| Urinary Bladder Wall | 5.79E-03 | 7.05E-03 |
| Uterus | 1.86E-04 | 1.35E-04 |
| Total Body | 1.27E-02 | 7.37E-03 |

**Supplementary Table 3.** Residence time (MBq-h/MBq) of source organs in each patient acquired using reference adult models in OLINDA/EXM 2.0.

| Organs | P1 | P2 | P3 | P4 | P5 | P6 | Mean | SD |
| --- | --- | --- | --- | --- | --- | --- | --- | --- |
| Heart Contents | 0.006 | 0.005 | 0.006 | 0.011 | 0.011 | 0.016 | 0.009 | 0.004 |
| Kidneys | 0.008 | 0.011 | 0.020 | 0.010 | 0.015 | 0.014 | 0.013 | 0.004 |
| Liver | 0.014 | 0.012 | 0.030 | 0.017 | 0.016 | 0.018 | 0.018 | 0.006 |
| Lungs | 0.032 | 0.028 | 0.036 | 0.060 | 0.027 | 0.038 | 0.037 | 0.012 |
| Pancreas | 0.004 | 0.001 | 0.002 | 0.004 | 0.002 | 0.002 | 0.003 | 0.001 |
| Red Marrow | 0.008 | 0.004 | 0.007 | 0.005 | 0.004 | 0.003 | 0.005 | 0.002 |
| Spleen | 0.002 | 0.002 | 0.004 | 0.005 | 0.002 | 0.004 | 0.003 | 0.001 |
| UB Contents | 0.036 | 0.050 | 0.342 | 0.042 | 0.102 | 0.040 | 0.102 | 0.120 |
| Uterus | 0.005 | 0.002 | 0.010 | / | / | / | 0.006 | 0.004 |
| Body Remainder | 0.686 | 0.686 | 0.339 | 0.638 | 0.631 | 0.523 | 0.584 | 0.134 |

**Supplementary Table 4.** Comparison of SUVmean between ^68^Ga-FAPI-04 (time point 6) and ^18^F-FDG PET/CT in organs of Chinese patiens.

|  | FAPI | | FDG | |
| --- | --- | --- | --- | --- |
| Organ | Mean | SD | Mean | SD |
| Parotid | 1.89 | 0.59 | 1.23 | 0.39 |
| Thyroid | 2.47 | 0.99 | 1.53 | 0.51 |
| Heart | 1.12 | 0.19 | 1.76 | 0.21 |
| Lungs | 0.49 | 0.06 | 0.36 | 0.08 |
| Liver | 0.63 | 0.08 | 2.23 | 0.26 |
| Gallbladder | 0.50 | 0.12 | 0.85 | 0.45 |
| Pancreas | 1.54 | 0.65 | 1.47 | 0.18 |
| Spleen | 0.75 | 0.10 | 1.69 | 0.24 |
| Kidney | 1.58 | 0.23 | 2.47 | 0.24 |
| Bone marrow | 0.41 | 0.08 | 1.61 | 0.27 |
| Bone Cortex | 0.52 | 0.50 | 0.45 | 0.24 |
| Stomach | 0.73 | 0.14 | 1.22 | 0.45 |
| Small intestine | 0.69 | 0.15 | 1.79 | 0.45 |
| Colon | 0.68 | 0.38 | 1.59 | 0.58 |
| Muscle | 1.28 | 0.21 | 0.56 | 0.07 |

**Supplementary Table 5.** SUVmax and SUR of ^68^Ga-FAPI-04 in lung cancer patient 1, patient 4 and patient 6.

| Patient 1 | SUVmax (lesion) | SUVmax (lung) | SUR |
| --- | --- | --- | --- |
| 0.2 min | 7.83 | 2.56 | 3.1 |
| 4 min | 13.32 | 1.35 | 9.9 |
| 12 min | 17.03 | 1.19 | 14.3 |
| 21 min | 16.76 | 1.08 | 15.5 |
| 32 min | 18.58 | 0.99 | 18.8 |
| 44 min | 17.62 | 0.86 | 20.5 |

| Patient 4 | SUVmax (lesion) | SUVmax (lung) | SUR |
| --- | --- | --- | --- |
| 0.2 min | 4.53 | 3.57 | 1.3 |
| 4 min | 7.95 | 1.84 | 4.3 |
| 12 min | 8.62 | 1.29 | 6.7 |
| 21 min | 8.31 | 1.7 | 4.9 |
| 32 min | 8.9 | 1.17 | 7.6 |
| 44 min | 9.74 | 1.11 | 8.8 |

| Patient 6 | SUVmax (lesion) | SUVmax (lung) | SUR |
| --- | --- | --- | --- |
| 0.2 min | 4.27 | 2.96 | 1.4 |
| 4 min | 5.43 | 2.44 | 2.2 |
| 12 min | 6.61 | 1.76 | 3.8 |
| 21 min | 6.62 | 1.28 | 5.2 |
| 32 min | 5.85 | 1.28 | 4.6 |
| 44 min | 5.64 | 0.95 | 5.9 |
